# Bioenergetics evolution: the link between Earth’s and Life’s history

**DOI:** 10.1098/rstb.2024.0102

**Published:** 2025-08-07

**Authors:** Anastasiia Padalko, Val Karavaeva, Jordi Zamarreno Beas, Sinje Neukirchen, Filipa L. Sousa

**Affiliations:** ^1^ Department of Functional and Evolutionary Ecology, University of Vienna Faculty of Life Sciences, Vienna, Austria

**Keywords:** energy metabolism, ATP, electron transport chains, substrate-level phosphorylation, metabolic diversity, modularity

## Abstract

The history of life intrigues both researchers and society, as it is human nature to question our origins. Our understanding of microbial evolution comes mainly from genomic data and geological evidence. Recent advances in sequencing technologies are revealing vast insights into microbial diversity, especially among uncultured lineages. While metagenomics indicates the existence of novel lineages, their ecological functions remain unknown. To unlock these mysteries, we need to shift focus from genomics to understanding their physiology. A barrier to understanding environmental microbes lies in our limited knowledge of their energy-harnessing and conservation strategies. Phylogenetic trees built from universal genes can group thousands of lineages but fail to capture the entire genome or reflect key physiological traits, especially with lateral gene transfer complicating evolutionary patterns. To deepen our knowledge of microbial evolution, a promising strategy combines large-scale comparative phylogenetic analyses of genes related to physiology with experimental data. Geochemical records of ancient energy sources can act as evolutionary constraints. This top-down approach would help rule out traits that could not be ancient, narrowing the physiological possibilities of early microbial life. Focusing on how microbes harnessed energy during evolution could bridge the gap between geochemistry and microbiology, providing testable predictions about bioenergetic transitions.

This article is part of the discussion meeting issue ‘Chance and purpose in the evolution of biospheres’.

## 1. Introduction

Life. Life as we know it is a far-from-equilibrium biochemical system of redox reactions; an extraordinary one, where recurrent domains and cofactors and a limited set of chemical elements and compounds are continuously recruited to sustain any given living entity. In short, *Life is* driven by *a chemical reaction that produces ATP*, with ATP maintaining the life process far from equilibrium. Modern microbes have two ways to conserve energy as ATP: chemiosmotic coupling [1] and substrate-level phosphorylation (SLP) [2]. Even if at the onset of life other energetic currencies might have been used [3–5], it is generally accepted that by the first emergence of microbial lineages, ATP had already obtained its central role in microbial bioenergetics [6,7], because both ATP as an energy currency and the rotor-stator ATPase are universal. This is furthermore corroborated at the protein level, by the structural similarities between the ATP synthases present in all domains of life [8,9]. There are also clear indications that chemiosmotic energy harnessing was already in place in ancient microbes [7,10–12], coexisting with simpler mechanisms of SLP, which are clearly also ancient [12]. Yet, the ways microbes explore (and strongly influence) the modern environment in terms of how they conserve their energy and obtain nutrients from the surroundings are highly diverse and still not fully understood.

Modern microbes play crucial roles in all of Earth’s global geochemical cycles, contributing to climate change [13], influencing greenhouse gas emissions [14], fixing carbon and nitrogen from inorganic sources [14] and producing O_2_ for us to breathe [15]. They have learned to use light [15], hydrogen [16], organic and inorganic forms of carbon [17,18], nitrogen [19] and sulfur [20–22] compounds and transition metals [23,24] as electron donors and/or electron acceptors to generate ATP. Some microbes also learned to use high-potential compounds, such as oxygen or nitric oxide as electron acceptors, while others (still) obtain their energy by using more reduced compounds as terminal acceptors, such as metals [25] or organic metabolites [26]. In many cases, more than one form of energy metabolism is present in an individual organism (facultative growth), each trait delivering different ATP yields. This remarkable versatility provides flexibility and robustness to microbial diversity and makes them fit to successfully adapt and survive in diverse conditions. From an evolutionary perspective, it is clear that all of the extant microbial diversity cannot have arisen at the same point in time, because the end-products of some pathways (in particular, O_2_ and terminal acceptors derived from O_2_) are needed for other pathways to evolve. There had to be a sequence of events. Hence, through microbial evolution, stepwise transitions from simpler to more complex energy-harnessing solutions must have existed. This brings us to a major question of this paper: can the functional and temporal connections of such events be identified?

Before the advent of genomics and phylogenies, (micro)biologists already addressed energy metabolic solutions that run the life process, based on their biochemical observations; that is, the inferences of what would or would not be suitable for a specific organism to grow, or what property (catalytic or biochemical) the specific enzymes had that enabled them to perform a given biological reaction. It was through those early studies that we first learned about *chemiosmotic coupling* and *substrate level phosphorylation* as ways to produce ATP, that the concept of *bioenergetics*—the way energy is transferred, transformed and transduced within a biological unit—was established [27]. From bioenergetics, we learned that microbes have breathtaking physiological (energy-conserving) diversity and can obtain energy by photosynthesis [15,28,29], respiration (chemiosmosis) [30] or fermentative processes [31,32]. Advances in physiology, however, always come at slower paces: it is one reaction, one gene, one pathway at a time.

We now live in the ‘*post-genomic era*’, approaching an *‘AI era’*, so the focus of the field has shifted. As technology progresses, it is now easy and affordable to sequence genes, genomes and environmental (meta)genomes— thousands of them at one go [33]. The computer power to handle such data has also kept up to pace so that we are now able to generate phylogenetic trees based on universal genes for thousands of lineages at a time [34]. The advances in sequencing techniques are overwhelming databases with rich and novel insights into *microbial taxonomic diversity*, in particular about new and (mostly) yet-uncultured lineages that thrive in fascinating and unforeseen environments. Mainly due to environmental sequencing, over the last two decades, the number of available archaeal and bacterial genomic records (assemblies) increased from around a mere three thousand to more than two million and our view in systematics had to expand [34] to accommodate more than 30 new taxa at roughly the highest rank (phylum).

Through metagenomics, we now know that those microbes are there, but (for most of them) we still do not understand what they are doing, or how they make a living. Somewhere along the way, the evolution of (energy) metabolism as a whole has almost been disbanded. While it is discussed in the context of last universal common ancestor (LUCA) [7,35,36], or in the context of current microbial diversity [37,38], perhaps with the exception of oxygen usage [39], the transition from one type of metabolism to the other is often omitted, with notable exceptions such as the case of a recent review [40] addressing how genomic information can be superimposed on geological and geochemical records to trace the progression of life and how this can be incorporated within the broader framework of biospheric evolution, considering key transitions in the chemical properties of the oceans, continents and atmosphere and the diversity and development of early metabolic processes, their integration with biogeochemical cycles and their role in the oxygenation of the early biosphere.

If we aim to understand microbial diversity and its evolution, we must focus on how microbes harness energy from the environment and conserve energy and how this has changed over time. This is where Earth’s history meets Life history, where metals and cofactors, their possible electron transfers and thermodynamic ranges, biological ‘*Lego’* blocks or redox kits, lateral gene transfer (LGT), pathway (re)arrangements and duplications events start playing an important role.

## 2. Looking back in time

Empirically there are two ways to look into early microbial evolution: geological records and genomes. Despite erosion and plate tectonic movements, geological records harbour evidence of biological processes [41,42]. They also give us clues regarding the type(s) of energy and compounds that might have been available through time to be used in biological activities. On the other side, genomes also record their own history. Microbial bioenergetics is a bridge between those two approaches.

## 3. Phylogeny, bioenergetics and lateral gene transfer

More than 45 years ago, the advances in molecular phylogenies and 16S rRNA gene sequencing provided the tool and the universal tree that microbial systematics had long been looking for: the rRNA tree installed the direly needed order into microbial systematics [43]. It is now state-of-the-art to use robust phylogenies based on rRNA genes or on approximately 35 nearly universal proteins involved in informational processing or ribosome biogenesis to place newly sequenced organisms close to their phylogenetic relatives, to delineate to *whom* they are more related to. There are good reasons to believe that the ribosome is inherited vertically in archaea and bacteria [44,45], and, undoubtedly, robust phylogenies based on universal genes are essential and necessary, especially to bring order to microbial systematics and get the relationships right at higher taxonomic ranks. Getting these relationships right, however, is not always straightforward. An example was the debate [43,44,46–51] regarding the *ancient* or *late* origin of eukaryotes or the two versus three domains of life discussion. In the *old* rRNA trees [43,44], eukaryotes were usually found as a sister group of archaea. With more sophisticated methods and based on concatenated sets of ribosomal proteins rather than rRNA, newer trees are systematically retrieving the so-called two-domain tree, where eukaryotes are no longer a sister group of archaea but are branching within this domain [46–51]. Opponents from both sides disputed which fraction of the data should be retained in the alignment and which model is better suited to infer the tree topology [47,48,52,53]. Although in this case the dispute seems to be almost settled, with the dominant view posing eukaryotes related to an ancestral Asgard-like host [50,51], trees are prone to change, and still-to-be-discovered lineages might, in the future, be closer to the eukaryotic ancestor. In any case, in terms of metabolic diversity, and, in comparison with archaea and bacteria, eukaryotes are not so exciting. As a domain, and with the exception of photosynthetic lineages that acquired this trait via an endosymbiotic event [54], eukaryotic bioenergetics is an extremely narrow sample of the archaeal and bacterial one [55], with diversity arising mainly through differential gene losses. That is to say, physiological diversity (energy conservation) is mainly an archaeal and bacterial feature, and eukaryotes almost do not bear upon the issue of physiological (energy-conserving) evolution, except perhaps the nature of the energy metabolism possessed by the host that acquired the mitochondrion [55–57] and the ancestral mitochondrion itself [54,58–61].

**Figure 1.**
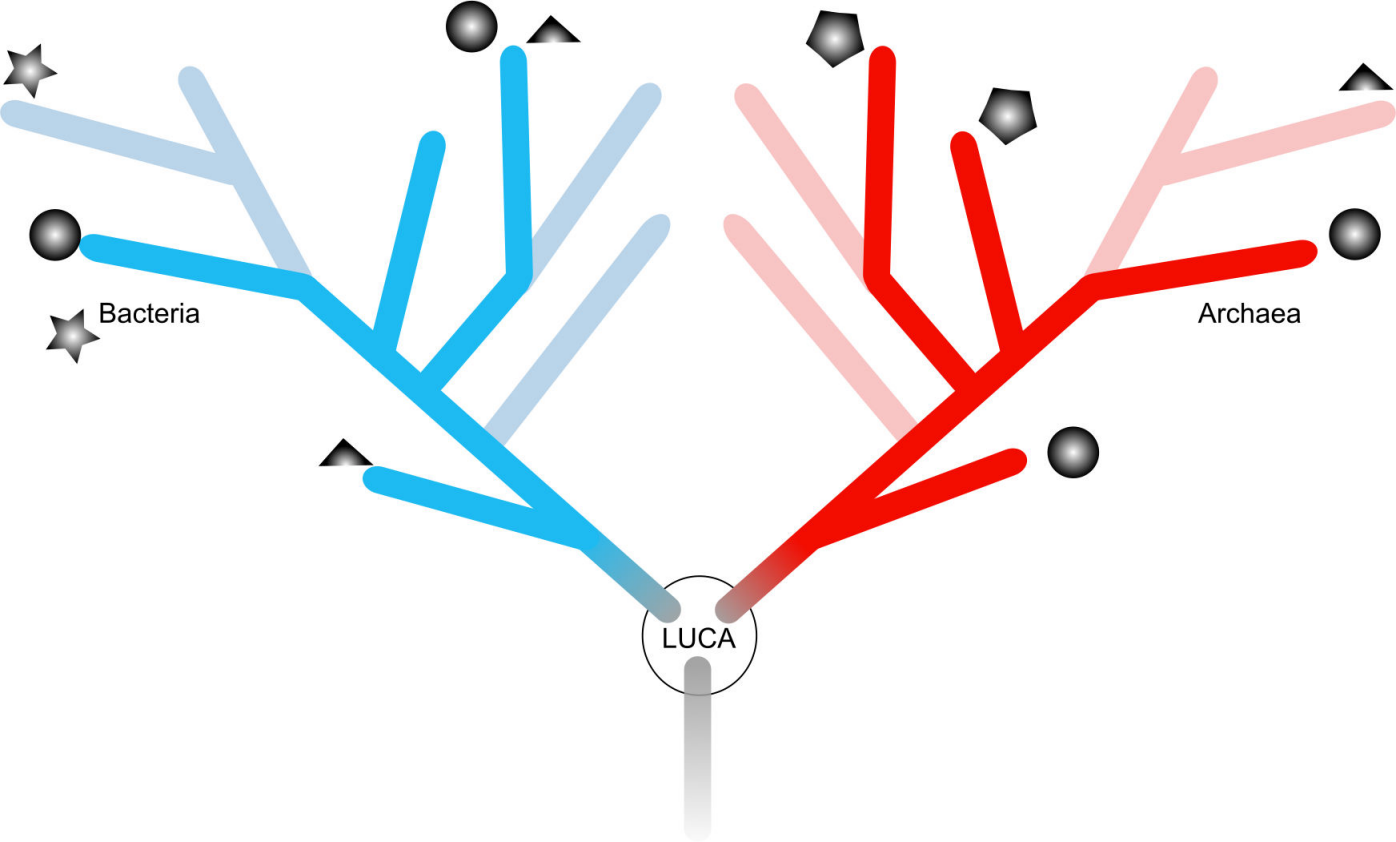
Distribution of energy metabolism (symbols) across a tree of life. Schematic representation with archaea shown in red and bacteria in blue where each symbol represents a hypothetical type of energy metabolism. Lighter-coloured branches represent extinct lineages. Adapted from Weiss *et al.* [7].

At this point, one might ask why not just construct the best tree we know how to construct and map the evolution of physiological traits across that tree, using the ribosomal protein dataset that everyone is currently using. In fact, many such attempts have already been reported, where gene distributions were plotted on a backbone phylogeny [62,63] that has been used to investigate gene distributions [64] over time. Such approaches are highly dependent on the assumed reference tree and on the evolutionary model(s) chosen, but more severely, physiological evolution in archaea and bacteria is marked by LGT events that have occurred over time and those might not be captured with these approaches (electronic supplementary material, figure S1). The widespread presence of a gene (or trait) across many distantly related genomes is not an indication *per se* of its ancestry (figure 1). Oxygen respiration, for example, has rapidly spread among all archaeal and bacterial lineages after the cyanobacterial origin of the oxic environment [29,65]. Moreover, backbone-tree-based approaches assume that genes within genomes tend to share the same evolutionary history. It is becoming increasingly clear that the evolution of microbial traits, the evolution of bioenergetics processes, is decoupled from the evolution of ribosomal lineages [66]. Both processes drive microbial diversity, but the two do not necessarily coevolve over the entire geological time. This is seen by the assembly of photosynthesis with three kinds of CO_2_ fixation pathways [15], the patchy distribution of sulfate reduction processes across archaeal and bacterial lineages [22,67] or even in the ways microbes learned to deal with arsenic. More recently, probabilistic tree reconciliation methods have been developed that can, in principle, in a fast manner reconcile systematics with metabolic evolution [68]. But even then, the modular and chimeric nature of life, as explained below, can complicate the analysis.

RNA-based trees capture important and valuable relationships regarding an important part of life: *systematics*. However, they fail to deliver information regarding how the rest of the genome has evolved. For the evolution of *bioenergetics*—the history of the essential processes for any microbe to exist—the issue is how energy-conservation machinery and strategies started and changed through time. The synthesis of RNA itself, as well as of all the universal ribosomal proteins used to construct such trees, are dependent on cell metabolism, on ATP for their synthesis [69]. Microbial bioenergetics, i.e. the way microbes gather their bulk ATP, is not encoded in any of the informational genes used for the tree of life reconstructions; it is usually encoded by a mere dozen or so (or in the case of photosynthesis *ca* 100 [28], in the case of methanogenesis *ca* 200 [70]) genes among thousands in the genome that, throughout time, were *passed* and *shuffled* around, crossing over and over again the domain boundaries of systematics [62,71] (whereby we wish to mention that methanogenesis in Archaea has probably never been transferred to bacteria and rarely transferred within the domain [72,73] based on current data, although it often has been replaced). Despite the massive sequence divergence that has been erasing part of their primordial signal (like erosion does to rocks), and despite all the possible LGTs they might have been subjected to (that introduce foreign material, reshape genomic architecture and foster genetic diversity in a manner comparable to how plate tectonic reshape landscapes, introduce new materials and drive geological diversity [74]), those genes and genomes still record their own history. Thus, to better understand the evolution of microbial energy metabolism, the *evolution of the genes specifically involved in and genetically associated with energy conservation* has to be taken into account in the light of microbial LGT.

## 4. Bioenergetics taken from Earth’s history

Earth too, like genomes, records its own history [42]. But throughout time, Earth’s history has been partially erased, and those parts are forever and irreparably lost. Yet, within the core of old rocks, we can still find information regarding Earth’s early environment and evidence for ancient microbial physiological traits [75,76] in the form of carbon [77], sulfur [78] and nitrogen [79] isotopes. In addition, rocks also provide indirect evidence for molecular O_2_ and changes in the redox potential of the environment. The relics of old microbial activities we still find today preserved in rocks tell us that it is microbial metabolism and not the evolution of their ribosomes that fits into the bigger picture of Earth’s history. Both microbial and Earth’s history have influenced (and still do) one another over time. As summarized by Olivia Judson, on a broader scale, Earth has gone through five ‘energetic epochs’ or energy expansions of evolution: geochemical energy, sunlight, oxygen, flesh and fire [80]. The first three are the ones that underpin almost all of the extant microbial diversity.

In a very reductionist view, we can summarize all physiological (energy-conserving) traits as sequences of electron transfers between environmentally available electron donors and acceptors, with the consequence of ATP formation. However, as Earth changed, the availability of electron acceptors and donors for biochemical processes also changed. Thus, we can take valuable lessons from Earth’s history regarding what forms of energy and compounds were available at a given point and how those have changed over time. If analysed in a systematic way, it will take us a step closer to the elucidation of how microbial physiological evolution has unfolded.

We now know that early Earth was very different from modern Earth [81–83], and consequently, the biochemical processes more favoured at that time were different from the ones preferred nowadays by microbial life. The oldest evidence of Life currently points to an early existence of biogenic carbonaceous and graphite material at approximately 3.7 Ga [75,76], after the time when the primordial liquid oceans are thought to have been formed [83]. Rocks also preserve evidence for N_2_ fixation as old as 3.2 Ga [77] or sulfate as around 3.5 Ga [78,84]. Early Earth is depicted to us by geologists as having a reductive environment, with chemical species naturally occurring in a more reduced state and where more oxidizing compounds, such as O_2_, were rare or even absent [40,85,86].

## 5. Bioenergetics, along rocks, along genomes

While geology runs into an impasse regarding the age of molecular dioxygen [87], biologists until recently did not: microbes only started to use O_2_ after the appearance of cyanobacteria and oxygenic photosynthesis. However, in the last years, several ‘O_2_ early’ scenarios have been proposed [88,89]. The evidence for the late incorporation of O_2_ in energy metabolism comes from observations of the peripheral role of O_2_ in biological networks [90] and the O_2_-dependent ramifications that occur in many pre-existing pathways [11,18,91]. Thus, oxygenic photosynthesis is not an ancestral process and, taking the last arguments into account, is an older process than aerobic respiration.

Besides oxygenic photosynthesis, very small amounts of O_2_ can be biologically produced by methane-oxidizing bacteria [92] or by chlorite dismutases [93]. However, it is not clear how old these enzymes are, nor their primordial substrate or direction, and what would have been the fate of the eventually produced O_2_ (if used for respiration, if promoted damage to the cells or if it escaped the cell and reacted with available chemical species, potentially changing locally the redox environment of the cells).

Until then, microbes were using other sources of energy, many with lower redox potential acceptors than O_2_, to obtain their ATP. This implies that the first lifeforms were anaerobes [94], and all biological processes directly and indirectly related to O_2_ are more recent microbial innovations. Most, but not all [89], biologists agree that oxygenic photosynthesis arose from anoxygenic photosynthesis [15,28,29]. However, the evolution of photosynthetic electron transport chains (with chlorophyll and/or other pigments) requires the previous existence of basic components, such as quinones and hemes, denoting that heme-dependent respiratory electron transfer chains (ETCs) preceded photosynthesis in evolution. In other words, anaerobic respiratory chains—of which there are many forms with many different terminal acceptors—are older than both photosynthesis and aerobic respiration. However, no further ‘easy’ insight can be obtained. In order to look further into the past, we need to look into all genomes and all known bioenergetic pathways, get the higher rank relationships right, gather extant and ancient geological records, construct all the relevant phylogenies, identify LGT, duplications and gene losses, and fill in the gaps. We also need to critically interpret the results and produce testable hypotheses. It is not enough to merely plot gene distributions onto a tree and, with arbitrary values of gains and losses, reconstruct all gene histories, having as basis other genes, which also have their own evolutionary history. The puzzle within microbial bioenergetics needs to be tackled by considering life’s property, its modularity and the independent evolutionary history of its individual pieces.

## 6. Connecting microbial metabolism and environment

The connection between the evolution of microbial metabolism and the geochemistry of its environment is nothing fundamentally new. Such connections have been reported in the past, either in the context of Life’s onset [8–12,85–98] or at a smaller scale, by connecting the evolution and diversification of a specific physiological trait with the geochemical history of Earth [99]. On the other hand, there are reports, with or without geochemical considerations, on the evolution of an entire energy-conservation trait based on the phylogenetic analysis of ‘a’ protein family or of ‘a’ gene considered to be essential for the process [100]. However, these approaches do not consider in a systematic way the full breadth of genomic space, the architecture of the complexes and all possible pathway organizations. There is a clear gap between ‘biochemistry/bioenergetics’ and ‘(meta)genomics’, a gap that needs to be filled. In this endeavour, one thing is very important, and it might sound trivial but it is not: some of the most crucial information that genomes contain about the evolution of metabolism is not where the genes are, which is readily seen from simple BLAST-and-tree type pipelines, but where they are missing, which requires a more systematic approach to the problem, with clustering and plotting of all presences and absences across very large all gene versus all genomes matrices [56,67] and using high-quality datasets (complete or almost complete with low contamination and further filtering). On the other hand, considering types and subtypes within the same protein family, each one, with its slightly different *in vivo* reactions, has to be incorporated within computational analysis, so a proper interpretation of phylogenies can be performed. The link between phylogeny, biochemistry and geochemical data can be challenging, mainly due to the incompleteness of the data (we do not have all rocks from all epochs, all activities from all enzymes, nor do we have all genomes from all environments). However, (micro)biological considerations can fill many of the existing gaps in our present knowledge. To acquire a more complete understanding of the evolution of energy metabolism, three main aspects need to be considered:

### (a) Mosaic nature of bioenergetic complexes

**Figure 2.**
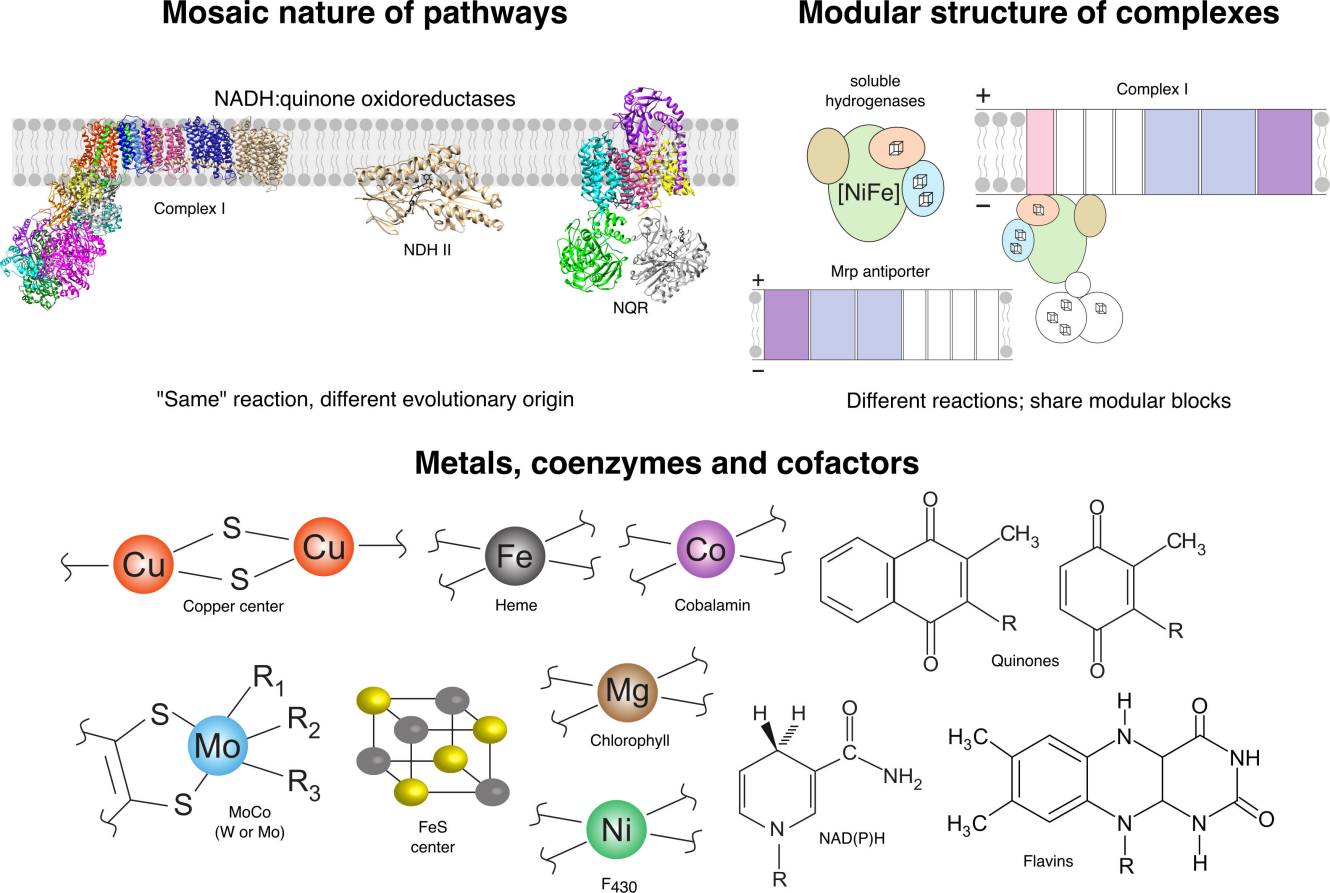
Modularity in bioenergetics. Left upper panel shows the non-homologous replacement of enzymatic complexes that perform similar functions within the cells (complex I [112], NDH II [113], NQR [114]). Right upper panel: reshuffling of protein modules to form complexes with a different function. Bottom: structures of cofactors used in metabolism.

It is part of human nature to name things and produce large lists and catalogues where items are grouped according to some common property. However, microorganisms are unaware of our naming or conventions. Their ‘goal’ in life, if it can be called that, is to adapt to their surroundings and survive. We have become accustomed to the idea of viewing physiological processes as a sequence of well-defined reactions, performed by specific enzymatic complexes that occasionally, in some ‘strange’ microbes, are replaced by some other complex or complexes. Through the hard work of many microbiologists, and sometimes also through metagenomics, we are discovering (or predicting) unexpected or hybrid pathway organizations, for example, in deep thermal aquifers of the West-Siberian Megabasin [101], hypersaline alkaline lakes [102] or alkaline soils [103], or even in the human gut [104]. Previously unknown reaction mechanisms, such as flavin-based electron bifurcation/confurcation [105,106], RuBisCO being an ancestrally heterotrophic enzyme involved in RNA fermentations [107–110], or old enzymes performing new reactions [111], do not fit with views of bioenergetics that were current only 15 years ago and call for a systematic analysis regarding ‘modularity and chimerism’ within physiological solutions (figure 2). Modular chimerism is present also in well-studied energy-harnessing systems, such as aerobic respiratory chains. The canonical (mitochondrial) version is composed of four multisubunit complexes: complex I (NADH dehydrogenase), II (succinate dehydrogenase), III (*bc*_1_ complex), IV (heme-copper oxygen reductase) and two electron carriers: ubiquinone (UQ) and a soluble cytochrome *c*. Contrary to mitochondria, archaeal and bacterial aerobic respiratory chains are branched, and microbes evolved non-orthologous alternatives for most of these complexes: complex I can be replaced by a type II NADH dehydrogenase (NDH II) [115] or by the NADH:quinone reductase (NQR) [114] (figure 2, left upper panel). Some microbes use the alternative complex III [116] instead of complex III to receive electrons from the quinol pool, while in others, the final reduction of O_2_ to water is performed by a *bd* oxidase [117] or by an alternative oxidase AOX [118]. Frequently, more than one enzymatic solution is present in the same organism, providing them with redundancy, robustness and versatility to deal with O_2_. In addition, archaea and bacteria are not limited to the use of UQ, and other quinones, with very different redox potentials— thus, very different energy yields—can be coupled to the electron transfer reactions [119]. Moreover, many microbes gain the ability to deal with O_2_ by simply acquiring (by LGT) one of the three terminal oxidases and coupling it to a pre-existing (anaerobic) ETC. This is the case of the *Acidianus ambivalens* archaeon that, in aerobic conditions, couples the oxidation of S^0^ to sulfate with the reduction of O_2_ using an HCO to catalyse the final reaction [120]. In addition, the same set of protein modules is used and reused over time to form complexes with different architectures or adaptations to perform different functions (figure 2, right upper panel).

This high diversity shows that, with the exception of a few traits, it is no longer enough to look just to one marker gene to predict how an organism gets its energy—it is necessary to perform a much larger reconstruction of their energy-conservation pathways. Even then, the direction in which the enzyme or pathway best operates *in vivo* is not always discernable, as in the case of many archaea containing the Wood–Ljungdahl pathway that can work in the oxidative and reductive direction [121]. In these cases, experimental characterizations and/or cultivation can settle the argument. Unfortunately, it is not feasible, in realistic time, to have all the data for all genes present in all genomes, nor grow all yet-uncultured organisms. This issue can be (at least, partially) bypassed with the cross-link of environmental information and the microbe genomic content, ideally combined with *in situ* proteomics, as several successful cases can attest [122,123].

### (b) ‘Lego’ structure of bioenergetics complexes

Despite the extraordinary diversity of life, a strikingly small set of core machinery is responsible for the different microbial bioenergetic solutions. Considering just oxidoreductases (EC1), Jelen *et al.* [124] estimated that a set of 400 homologous genes, shared by the three processes microbes currently use to obtain their energy (photosynthesis, respiration (chemiosmosis) and fermentation), are part of the complexes directly involved in electron transfer for energy metabolism.

Thus, the mosaic structure of life is not restricted to the way enzymes or complexes are sequentially organized within a given pathway. It is also present at a lower hierarchical level, in the way enzymatic complexes are assembled from a finite set of modules [125,126]. A clear example is the case of HCOs that are evolutionarily related to nitric oxide reductase, the enzyme that reduces NO to N_2_O in one of the last denitrification steps [127]. This illustrates that the same biological module was recruited, duplicated and/or adapted to participate in two distinct physiological processes: aerobic respiration and denitrification [88,128]. More intricate examples of this modular promiscuity can be inferred by the homologous relationship of some subunits from complex I, hydrogenases and antiporters [129] (figure 2, right upper panel), the alternative complex III and the large complex iron–sulfur molybdoenzyme protein family [126], or the heterodisulfide reductases present in methanogens and the quinone-interacting membrane-bound oxidoreductase complex from sulfate reducers [22,130]. These examples clearly indicate that, in order to gain the ability to deal with a new metabolite, microbes often recycle known building blocks to assemble complexes with novel architectures. Thus, to get a fuller picture of microbial metabolism evolution, the modular nature of bioenergetic complexes needs to be dissected, and the individual evolution of its individual components understood. This analysis will also allow addressing of questions regarding modular ancestry, such as: are promiscuous modules (the ones more frequently recruited to catalyse different reactions) older than the ones with a lesser functional diversity? And if so, how can the primordial activity be determined? For example, the FAD (Flavin adenine dinucleotide) subunit of succinate dehydrogenases/fumarate reductases (complex II, Sdh/Frd) can be classified into six different types (A–E) and belongs to a broader family of proteins [131]. This family includes single enzymes like NADH reductases, L-aspartate oxidase and subunits from complexes such as thiol:fumarate reductases, anaerobic glycerol-3-phosphate dehydrogenase and adenosine 5-phosphosulfate reductase [131]. It has been suggested that this subunit could date back to LUCA, the last universal common ancestor of all life forms [35]. However, a large phylogenetic study of Sdh/Frd across a dataset of 35 000 assemblies revealed that most types are not monophyletic, with archaeal and bacterial sequences appearing in multiple clades [132]. For instance, the well-characterized Sdh type E from *Sulfolobus solfataricus* [133,134] is as closely related to fumarate reductase from *Escherichia coli* as it is to the soluble thiol:fumarate reductases from methanogens, which is not involved in ETCs nor in the TCA cycle. This can lead to issues in functional annotations, and, in fact, several thiol:fumarate reductases are incorrectly annotated as Sdh in the *Kyoto Encyclopedia of Genes and Genomes* [132]. Therefore, incorporating synteny and complex completeness into the analyses is essential. However, this level of integration is currently difficult to achieve in computational analyses without scripting and manual inspection of phylogenies, which might be unfeasible in the case of large-scale analysis.

### (c) No enzyme (or pathway) is an island: metals, prosthetic groups and coenzymes

This brings us to the last point: how does bioenergetics operate? Enzymes that contain transition metals, iron–sulfur centres, flavins, hemes or other prosthetic groups catalyse the majority of bioenergetically relevant reactions (figure 2, bottom). These cofactors can simply act as electron transfer sites or have an essential role in catalysis [135]. Many of these complexes can donate (or receive) electrons directly to other proteins or, instead, transfer them to coenzymes, such as NADH or quinones. The use of cofactors in many biological processes, their universal conservation and their proven catalytic potential make them essential for any living system—without them, no organism can survive, as the investigations of autocatalytic sets in archaeal and bacterial metabolic networks clearly show [136–138]. Although with slight differences in methodology, those studies showed that the removal of cofactors and/or coenzymes from the network had a dramatic effect on the size of the recovered network. Thus, a thorough analysis regarding the possible availability of cofactors and coenzymes, also considering the evolution of their biosynthetic routes, is critical in the framework of microbial evolution. Interestingly, the first two studies identified ATP as an autocatalytic compound indispensable to kick-start metabolism, bringing us back to where this article started: microbial evolution and diversity are fuelled by the quest for ways to make ATP.

## 7. Conclusions

**Figure 3.**
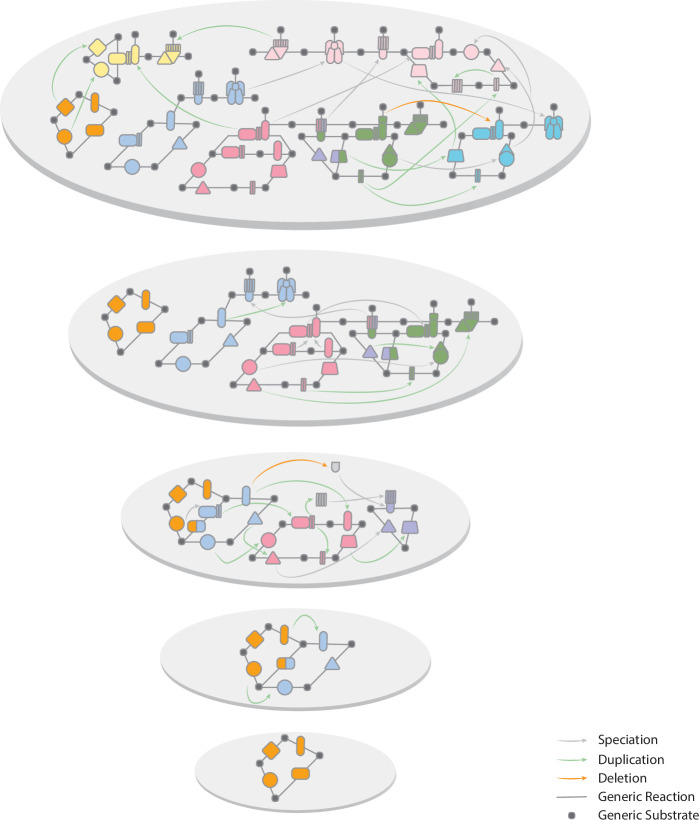
Stepwise bioenergetics evolution: each grey circle represents a snapshot of the entire biological energy metabolism diversity, at a given time. Proteins are coloured by type of energy metabolism with compounds represented as small dark grey circles. Proteins with the same shape are homologous. Grey full lines represent generic reactions and origin by speciation, and duplication is represented by grey and green arrows. Events of losses are indicated by an orange arrow.

A natural link between Earth’s history and Life’s history is bioenergetics. Bioenergetics is the connection that joins two broad scientific fields: geochemistry and microbiology. To systematically elucidate the evolution and diversity of physiological traits, the functional and temporal connections between them and to create snapshots of archaeal and bacterial physiological diversity over time (figure 3), bioenergetics evolution needs to be tackled.

This endeavour requires interdisciplinary approaches that integrate phylogenies and bioinformatic screenings with, for example, biochemistry. Moreover, this could foster collaborations between bioinformaticians and experimentalists and has the potential to lead to further experimental characterizations and to produce better and more accurate predictions. Successful examples include the investigation of the evolutionary link between L-malate and L-lactate dehydrogenases, with the discovery and characterizations of a group of intermediary sequences exhibiting mixed functional properties [139], or the discovery of a new iron–sulfur centre assembly pathway coupled with the evolutionary history of the different iron–sulfur centre assembly pathways [140].

By approaching the evolution of physiology in a fundamentally modular manner, with respect to (i) proteins promoting energy-conserving redox reactions (and their different subtypes), (ii) their cofactors, (iii) their phylogenetic presence (*and absence*) in the temporal context of (iv) the progression of redox midpoint potentials from low (ancestral H_2_-dependence) to high (modern O_2_-dependence) during Earth history on the basis of (v) genomic and metagenomic information across modern environments, (vi) coupled with experimental characterizations of enzymes, we predict that progress can be made in understanding how extant metabolic diversity arose in a stepwise fashion, from a simpler universal ancestor.

## Data Availability

All data are provided electronically as supplementary material [141].
